# Is *Galba schirazensis* (Mollusca, Gastropoda) an intermediate host of *Fasciola hepatica* (Trematoda, Digenea) in Ecuador?

**DOI:** 10.1051/parasite/2017026

**Published:** 2017-06-30

**Authors:** Yannick Caron, Maritza Celi-Erazo, Sylvie Hurtrez-Boussès, Mannon Lounnas, Jean-Pierre Pointier, Claude Saegerman, Bertrand Losson, Washington Benítez-Ortíz

**Affiliations:** 1 Parasitology and Pathology of Parasitic Diseases, Fundamental and Applied Research for Animals & Health (FARAH), Faculty of Veterinary Medicine, University of Liège Quartier Vallée 2, 6 Avenue de Cureghem 4000 Liège Belgium; 2 International Center for Zoonosis, Day Hospital, Central University of Ecuador PO Box 17-03-100 Quito Ecuador; 3 MIVEGEC, UMR IRD 224-CNRS 5290-UM 911 Avenue Agropolis 34394 Montpellier France; 4 Department of Biology Ecology (Sciences Faculty), Montpellier University 2 Place Pierre Viala 34060 Montpellier France; 5 PSL Research University: EPHE-UPVD-CNRS, USR 3278 CRIOBE, Perpignan University 58 Avenue Paul Alduy 66860 Perpignan France; 6 Research Unit of Epidemiology and Risk Analysis Applied to Veterinary Sciences (UREAR-ULg), Fundamental and Applied Research for Animals & Health (FARAH), Faculty of Veterinary Medicine, University of Liège Quartier Vallée 2, 7A-7D Avenue de Cureghem 4000 Liège Belgium; 7 Veterinary Medicine and Zootechny Faculty, Avenida América, Central University of Ecuador PO Box 17-03-100 Quito Ecuador

**Keywords:** *Fasciola hepatica*, *Galba schirazensis*, Multiplex PCR, Ecuador, Epidemiology

## Abstract

Fasciolosis is a widely distributed disease in livestock in South America but knowledge about the epidemiology and the intermediate hosts is relatively scarce in Ecuador. For three months, lymnaeid snails were sampled (*n* = 1482) in Pichincha Province at two sites located in a highly endemic area. Snails were identified (based on morphology and ITS*-*2 sequences) and the infection status was established through microscopic dissection and a multiplex polymerase chain reaction (PCR)-based technique. Techniques based on morphology were not useful to accurately name the collected snail species. Comparison with available DNA sequences showed that a single snail species was collected, *Galba schirazensis.* Live rediae were observed in 1.75% (26/1482) and *Fasciola* sp*.* DNA was detected in 6% (89/1482) of collected snails. The COX-1 region permitted identification of the parasite as *Fasciola hepatica*. The relative sensitivity and specificity of the microscope study, compared to PCR results, were 25.84% and 99.78%, respectively. The mean size of the snails recorded positive for *F. hepatica* through crushing and microscopy was significantly higher than the mean size of negative snails, but there was no such difference in PCR-positive snails. The role of *G. schirazensis* as an intermediate host of *F. hepatica* in Ecuador is discussed and the hypothesis of an adaptation of the parasite to this invasive snail is proposed. For the first time, an epidemiological survey based on molecular biology-based techniques assessed the possible role of lymnaeid snails in the epidemiology of fasciolosis in Ecuador.

## Abstract (fr)

La fasciolose est une maladie largement distribuée chez le bétail en Amérique du Sud, mais les connaissances sur l’épidémiologie et les hôtes intermédiaires sont relativement rares en Équateur. Pendant trois mois, les Lymnaeidae ont été échantillonnés (*n* = 1482) dans la province de Pichincha dans deux sites situés dans une zone fortement endémique. Les mollusques ont été identifiés (en fonction de la morphologie et des séquences ITS-2) et l’état de l’infection a été établi grâce à une dissection microscopique et une technique de PCR multiplex. Les techniques basées sur la morphologie n’ont pas été utiles pour identifier avec précision l’espèce de mollusque récoltée. Une comparaison avec les séquences d’ADN disponibles a montré qu’une seule espèce de mollusque a été récoltée, *Galba schirazensis*. Parmi les mollusques collectés, des rédies vivantes ont été observées chez 1.75 % (26/1482) et de l’ADN de *Fasciola* sp. été détecté dans 6 % (89/1482). La région COX-1 a permis d’identifier le parasite comme *Fasciola hepatica*. La sensibilité relative et la spécificité des études microscopiques, en comparaison avec les résultats de la PCR, étaient respectivement de 25.84 % et 99.78 %. La taille moyenne des mollusques considérés positifs pour *F. hepatica* par écrasement et microscopie était significativement plus élevée que la taille moyenne des mollusques négatifs, mais une telle différence n’a pas été trouvée chez les mollusques PCR-positifs. Le rôle de *G. schirazensis* en tant qu’hôte intermédiaire de *F. hepatica* en Équateur est discuté et l’hypothèse d’une adaptation du parasite à ce mollusque invasif est proposée. Pour la première fois, une enquête épidémiologique, basée sur des techniques de biologie moléculaire, a évalué le rôle possible des mollusques Lymnaeidae dans l’épidémiologie de la fasciolose en Équateur.

## Introduction

Fasciolosis is an important cosmopolitan parasitic disease, mainly of domestic ruminants [52] and man [28] with high potential risks of emergence or re-emergence [30, 57]. In South America, it is caused by a digenean trematode (Platyhelminth): *Fasciola hepatica,* which has a worldwide distribution. It causes severe outbreaks in livestock [54] and remains a true public health and veterinary problem, inducing important economic losses. South America is a region where human fasciolosis is considered as emergent and where the highest human prevalences of this disease have been observed [23, 27, 28]. Several cases of infection have also been reported in livestock, with important economic losses. For instance, in Argentina in 2009, bovine liver condemnation due to *F. hepatica* in the slaughterhouse of Atílio Vivácqua concerned 28.24% of the carcasses (9568/33,870) that led to approximately US $132,000 of losses [10]. In Peru, Espinoza and colleagues [22] estimated the losses due to liver condemnation to be around US $50 million.

Despite the recognition of fasciolosis as a threat in South America, epidemiological data for both humans and livestock remain scarce in Ecuador. For instance, only a few studies have dealt with fasciolosis and it is currently considered that prevalences are low (e.g., [33]), although the human population at risk was estimated at 20.6% [30]. The presence of human fasciolosis in Ecuador was reported for the first time in the international scientific literature in 2000 [55], when 6% (9/150) of an Andean community were found to be seropositive for *F. hepatica*. A coprological study in this country showed a prevalence of 0.5% in a group of children [24]. In 2005, in the municipal slaughterhouse of Machachi (Ecuador), 12.28% (162/1319) of bovine livers were condemned due to fasciolosis [21]. Further studies are therefore needed to evaluate the risk of fasciolosis in Ecuador.

The life cycle of *F. hepatica* involves a mammalian definitive host (domestic and wild ruminants, pigs, rodents, humans) and a molluscan intermediate host (Mollusca: Lymnaeidae) [5]. *Fasciola hepatica* adolescaria from the snail encyst on surfaces in water. Thus, a good knowledge of intermediate hosts and their prevalences is required to identify areas and/or periods with epidemiological risks. Conventional wisdom has said that, apart from *Lymnaea rupestris*, for which the infection by *F. hepatica* had never been clearly demonstrated, seven species may act as potential vectors of fasciolosis in the Neotropics [8, 9, 16, 17, 32]: *Lymnaea diaphana*, *Pseudosuccinea columella*, *Galba cousini*, *G. viator*, *G. cubensis*, and *G. truncatula*. Recently, Correa and colleagues [16] unambiguously showed the presence of another true species they called *Galba* sp. Further analyses showed that this species is similar to *Galba* (*Lymnaea*) *schirazensis* [8]. Due to phenotypic plasticity in shell shape [50] and extremely homogenous anatomical traits among species [17], the correct identification of all these species is impossible in the “truncatula-like group”, i.e., phenotypically similar species: *Galba truncatula*, *G. schirazensis*, *G. viatrix*, and G*. cubensis*. In fact, the combination of several anatomical parameters of the reproductive system is of no use to discriminate these different species [17, 43], although DNA-based approaches (barcoding) unambiguously ascribe individuals to one species or another. In Ecuador, three lymnaeid species have been reported: *G. cousini* [40, 41], *P. columella* [37, 41], and *G. schirazensis* [8]. Only *G. cousini* was so far recorded to be naturally infested with *F. hepatica* in this country [58] with a very high prevalence of 31.43% (22/70). Neither *P. columella* nor *G. schirazensis* were described as infected in Ecuador, despite the fact that *P. columella* was recently found naturally infected in Brazil [15], Argentina [45], and Cuba [25], and that *G. schirazensis* from Colombia seemed able experimentally to harbor some larval stages of *F. hepatica* [20]. The detection of *Fasciola* sp. in the intermediate host is possible through different techniques [13]. Generally, microscope techniques are chosen in the developing countries because these methods are cheap, but this could lead to an underestimation of prevalence or false identification of parasite larval forms. Only one study used a molecular-based technique to assess the infection status in field-collected lymnaeids in South America (Argentina) [18]. The aim of the present study was to provide new insight into the epidemiology of *F. hepatica* in Ecuador especially regarding lymnaeid species, in an area where the intermediate and definitive hosts interact and adapt themselves in a permanent manner. For this purpose, we monitored the lymnaeid infection status in a three-month longitudinal survey and searched for the presence of *Fasciola hepatica* larvae by microscopic detection and DNA analysis.

## Materials and methods

### Sampling location

Snails were sampled in two pastures belonging to a private farm called “La Fontana” located in Ecuador, near Machachi (Province of Pichincha, in the county of Meija). The first sampled site (site 1) (0°26′37.59′′S; 78°32′24.12′′W, 2794 m above sea level (a.s.l.)) and the second sampled site (site 2) (0°26′30.66′′S; 78°32′38.21′′W, 2781 m a.s.l.) were wet pastures regularly grazed by cattle belonging to the farm. Data concerning general information about the farm, cattle management, and fasciolosis history were also collected.

A total of 184 Holstein Jersey cattle producing milk and 13 horses belong to this private farm, with 1200 ha of pastures. Pasture rotation took place every 2 months. Both sites were approximately separated by 1000 m and were crossed with small brooks (“acequias”) with slow flow (without connection between sites) and the snails were sampled all along the mud edge. This is the only source of water for the cattle in the field. The soil is volcanic in origin and vegetation was mainly composed of ray-grass (*Lolium* sp.) and aquatic plants. The farm had a long history of fasciolosis, as *F. hepatica* eggs were regularly observed during copro-parasitology. Approximately 50% of the animals were positive. Cattle were treated every three months with injectable nitroxynil (Nitromic^®^).

### Snail sampling

Snails (>4 mm) were collected every 2–3 weeks, between April and July 2013 (seven times), at both sites, for 30 min each, kept alive in plastic containers and transported to the International Center for Zoonosis in Quito for further analysis. The 1482 sampled snails were quickly sorted according to genus identification before anatomical examination. The height of the shell of each sampled snail was measured from the apex to the anterior margin. For identification purposes, three snails were randomly selected half the time (4/7) for each biotope (2) and the same snails were identified following anatomical dissection and molecular analysis (total 24).

### Snail identification

#### Snail processing, fixation, and morphological analysis

Snails collected in the field were dipped in an isoflurane (Iso-Vet) solution (one drop in 5 mL of tap water). They were then killed by plunging into hot water at 70 °C for about 40 s and transferred into cold water. Each specimen was gently pulled by the foot with a tweezer to disconnect the columellar muscle from the shell. The whole animal was drawn out of the shell and fixed in modified Railliet-Henry’s solution (distilled water 930 mL, sodium chloride 6 g, formalin (40%) 50 mL, acetic acid 20 mL) for anatomical examination. Several anatomical descriptions [36–41, 50] were used to identify species.

#### Sequencing reaction and alignment

A polymerase chain reaction (PCR) assay was used to amplify the ITS-2 rDNA sequence specific for lymnaeids (500–600 bp) [14]. The primers used were News2 5′-TGT-GTC-GAT-GAA-GAA-CGC-AG-3′ and Its2Rixo 5′-TTC-TAT-GCT-TAA-ATT-CAG-GGG-3′ [3, 6]. The sequences were amplified using a commercial kit (Taq PCR Master Mix, Qiagen) in a total volume of 25 μL (containing 3 mM of MgCl_2_ and 400 μM of each dNTP) in a Peltier Thermal Cycler (MJ Research) with an initial denaturation step at 94 °C for 3 min, followed by 40 cycles, each comprising denaturation at 94 °C for 30 s, annealing at 55 °C for 30 s, extension at 72 °C for 45 s, and a final extension step at 72 °C for 5 min. ITS-2 rDNA sequences were then purified using MSB Spin PCRapace (Invitek). Cycle sequencing reactions were performed (in triplicate and in both directions) by BigDye Terminator v3.1 (3730 DNA analyzer; Applied Biosystems) by GIGA Genomics Facility (Liège University, Belgium). Both strands of each sample were compared and used to reconstruct a consensus sequence. This was done in triplicate for each sample. Consensus sequences were made according to the results of sequencing of the PCR products and were aligned using BioEdit 7.1.10 [26] and analyzed using BLASTN 2.2.26 searches in GenBank (http//www.ncbi.nlm.nih.gov/BLAST). The species identity of sequences (144) obtained from PCR products was determined according to the highest BLAST match (with a threshold of 99–100% similarity).

### Prevalences

#### Snail crushing

To assess the *Fasciola* sp. infection status, the collected snails were squashed between two microscope slides and carefully examined under a microscope (×10 magnification). Larval forms (sporocysts, rediae, and cercariae) of *Fasciola* sp. were identified following identification keys [51]. The squashed body of each snail was then delicately recovered and put in an individually annotated tube for molecular analysis.

#### DNA extraction and pooling

Snail DNA extraction was based on the Chelex^®^ method, as previously described [14]. Briefly, the snail was mechanically disrupted with the help of a pellet mixer (Trefflab) in 100 μL of Chelex^®^ 5% (BioRad) and incubated for 1 h at 56 °C and 30 min at 95 °C in a Peltier Thermal Cycler (Techne TC). The mixture was centrifuged at 13,000 × *g* for 7 min. The supernatant was collected and stored at −20 °C until further analyses.

In order to reduce the number of PCRs to detect Fasciola DNA, pools of individuals were formed by mixing together 1 μL of each DNA sample with a maximum of 10 snails per pool. This mixture was considered undiluted. One microlitre of the mixture was then tested in the multiplex PCR described below. In case of pool positivity, snails were individually analyzed with the same technique.

The absence of internal control amplification (PCR inhibitors) for a pooled or an individual sample was assessed through 1/10 and 1/100 dilutions. Furthermore, the addition of 0.05% bovine serum albumin (BSA) to the PCR mixture at 1/10 dilution was tested for samples with absence of internal control amplification whereupon negative samples were definitively excluded from the study.

### Parasite identification

#### Multiplex PCR

A multiplex PCR assay [14] was used to amplify a highly repeated 124 bp sequence (microsatellite) specific for *Fasciola* sp. [29] and ITS*-2* rDNA sequence specific for lymnaeids (500–600 bp). The ITS-*2* sequence of the snail acts as a PCR internal control as its absence indicates potential presence of PCR inhibitors. The primers used for amplification of *Fasciola* sp. sequences were Fsh1 5′-GAT-CAA-TTC-ACC-CAT-TTC-CGT-TAG-TCC-TAC-3′ and Fsh2 5′-AAA-CTG-GGC-TTA-AAC-GGC-GTC-CTA-CGG-GCA-3′ and for lymnaeids ITS*-*2 amplification sequences were News2 5′-TGT-GTC-GAT-GAA-GAA-CGC-AG-3′ and Its2Rixo 5′-TTC-TAT-GCT-TAA-ATT-CAG-GGG-3′ [3, 6]. The sequences were amplified using a commercial kit (Taq PCR Master Mix, Qiagen) in a total volume of 25 μL in a Peltier Thermal Cycler (MJ Research) with an initial denaturation step at 95 °C for 5 min, followed by 40 cycles, each comprising denaturation at 95 °C for 1 min, annealing at 56 °C for 1 min, extension at 72 °C for 1 min, and a final extension step at 72 °C for 10 min. The amplification products were electrophoretically resolved in 2% agarose gels and stained with GelRed (Biotium). The limits of detection and specificity of this multiplex PCR were examined in a previous study [14].

#### Molecular parasite identification

Another PCR assay was used to amplify a 405 bp region of the cytochrome c oxidase subunit 1 gene (COX*-1*) to identify the species of fluke involved in the infected snails. The primers used were FhCO1F 5′-TAT-GTT-TTG-ATT-TTA-CCC-GGG-3′ and FhCO1R 5′-ATG-AGC-AAC-CAC-AAA-CCA-TGT-3′ as previously described [18]. The sequences were amplified using a commercial kit (Taq PCR Master Mix, Qiagen) in a total volume of 25 μL in a Peltier Thermal Cycler (MJ Research) with an initial denaturation step at 94 °C for 3 min, followed by 30 cycles, each comprising denaturation at 94 °C for 60 s, annealing at 56 °C for 60 s, extension at 72 °C for 60 s, and a final extension step at 72 °C for 10 min. COX*-*1 sequences were then purified using MSB-Spin PCRapace (Invitek). Cycle sequencing reactions were performed (in duplicate and in both directions) by BigDye terminator v3.1 (3730 DNA analyzer; Applied Biosystems) by GIGA Genomics Facility (Liège University, Belgium). Consensus sequences were made according to the results of sequencing of the PCR products and were aligned using BioEdit 7.1.10 [26] and analyzed using BLASTN 2.2.26 searches in GenBank (http///www.ncbi.nlm.nih.gov/BLAST). The species identity of sequences obtained from PCR products was determined according to the highest BLAST match (with a threshold of 99–100% similarity).

### Statistical analyses

To compare microscope and PCR results for the biotope considered, a Fisher’s exact test was used. The difference between the global prevalence as a function of time (date) was tested by a χ^2^ test. The distribution of size of snails based on the microscope or PCR results (positive versus negative) was assessed using a two-sample Wilcoxon rank-sum test. The relative sensitivity and specificity of the microscope related to the PCR results were estimated with the exact binomial distribution [42].

## Results

### Snail identification

A total of 1482 snails were collected: 1055 and 427 in the first and second sites, respectively.

#### Snail size and species morphological identification

The largest adult individual collected in the field was 6.2 mm in height (for the others, mean 4.83 ± *SD* 0.68 mm). The mean heights of the shell were calculated at site 1 (mean 4.32 ± *SD* 0.51 mm) and site 2 (mean 4.51 ± *SD* 0.68 mm). All the 24 examined snails belonged to small-shelled lymnaeids that are morphologically undistinguishable (“*truncatula*-like” *sensu* [17]). The observed phenotypic traits did not allow us to distinguish between *G. truncatula* [7, 50], *G. cubensis* [7, 50], *G. neotropica* [43], *G. viator* [7, 35], and *G. schirazensis* [8].

#### Sequencing reaction and alignment

**Figure 1.**
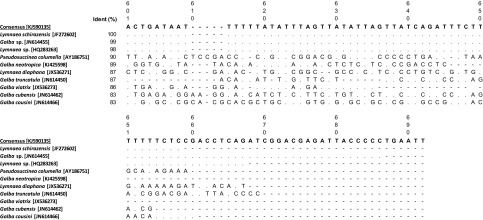
Sequence alignments of the ITS-2 sequences (5′–3′) of *Lymnaeidae* retrieved from the BLASTN analysis of *Lymnaeidae* found in South America. Ident is for Identity; dot (.) indicates conservation; hyphen (-) indicates alignment gap; a letter indicates substitution.

In order to accurately identify the collected lymnaeid species, ITS-2 sequences of 24 snails were examined. All the sequences (144) were identical. This sequence [GenBank: KJ590135] was found to be 100% identical to *L. schirazensis* [GenBank: JF272602]. Figure 1 shows the sequence alignments of the consensus sequence and the 10 first ITS-2 sequences retrieved from the BLASTN analysis of Lymnaeidae found in South America.

### Parasite prevalences

A global prevalence of 1.75% (26/1482) by crushing was calculated. No positive snail for *Fasciola* sp. was detected through crushing at site 2; the prevalence at site 1 was 2.46% (26/1055). Only rediae were observed in the infected snails (Table 1). Six percent (89/1482) of the collected snails contained DNA of *Fasciola* sp. This number corresponds to 8.15% (86/1055) at site 1 and 0.7% (3/427) at site 2. It was not possible to amplify the DNA of three snails collected at site 1. Therefore, those snails (0.2%) were excluded from the study (Table 1).

**Table 1.** Number of snails collected at sites 1 and 2 during the sampling campaign and infection status through microscopy and PCR.

Sampling date	25/4/13 = A	15/5/13 = B		29/5/13 = C		12/6/13 = D		3/7/13 = E		17/7/13 = F		24/7/13 = G	
Biotopes	1	1	2	1	2	1	2	1	2	1	2	1	2
Collected snails	110	71	40	141	64	188	36	111	134	314	65	120	88
Crushing positive snails (%)	9 (8.18)	6 (8.45)	0	5 (3.55)	0	5 (2.66)	0	1 (0.9)	0	0	0	0	0
PCR-positive snails (%)	14 (12.73)	11 (15.49)	0	9 (6.38)	1 (1.56)	15 (7.98)	0	10 (9.01)	2 (1.49)	19 (6.05)	0	8 (6.67)	0

### Molecular parasite identification

To assess the fluke species involved, 10.4% (9) of the 86 snails containing DNA of *Fasciola* sp. were randomly selected at site 1 and three snails containing DNA of *Fasciola* sp. at site 2 were added. The region of COX*-1* of the 12 samples was amplified and sequenced in duplicate and in both directions (48 sequences). All the sequences were identical but one. This sequence was found to be 99% identical to *F. hepatica* [GenBank: GQ121276] and 94% identical to *F. gigantica* [GenBank: GU112458].

### Statistical analysis

Fisher’s exact test was used to compare microscopy and PCR results for the site considered. Concerning site 1, the frequencies by date were not homogeneously distributed (*p* < 0.001) in function of the result obtained by crushing, whereas results obtained by PCR were homogeneously distributed (*p* = 0.10). The difference between the data obtained by crushing in function of the time (date) was tested by a χ^2^ test for site 1; prevalences for sampling dates A and B were significantly higher than for the other dates (χ^2^_ddl 1_ = 9.35; *p* = 0.002).

The distribution of size of snails in relation to the data obtained through microscopy or PCR at site 1 was assessed using a two-sample Wilcoxon rank-sum test. The size of the snails recorded positive for *F. hepatica* through crushing was significantly higher (*p* < 0.001) than that of negative snails. For PCR results, the size of the positive snails had no effect (*p* = 0.41).

The relative sensitivity and specificity of the microscope related to the PCR results were estimated with exact binomial distribution. The relative sensitivity and specificity were estimated to be 25.84% (CI 95%: 17.14–36.21) and 99.78% (CI 95%: 99.37–99.96), respectively. The κ coefficient was calculated as 0.38 (weak agreement).

## Discussion

### Detection and identification of parasites

The amplified 124 bp region of the parasite DNA was not sufficient to ascribe parasite species [31]; *Fasciola hepatica* was only identified according to the COX-1 region in infected snails. This study showed overall prevalences of 1.75% and 6% using microscopy and PCR, respectively. This difference is probably due to the low sensitivity of the crushing technique [13]. In a previous study, the infection rates of *F. hepatica* in *P. columella* were 17.5% and 51.3% by direct examination and PCR, respectively [18]. The relative specificity of the crushing method was very high (99.78%) as only three microscopically positive snails were negative by PCR. In contrast, the sensitivity was low (25.84%) because crushing overestimates the number of false negatives. This is because an infection with sporocysts is very difficult to record and the presence of shell fragments can hamper parasite detection. Furthermore, the very high sensitivity of PCR could overestimate the “true prevalence” as it detects specific DNA but does not give information about the viability of the parasites. Indeed, presence of snail DNA does not mean cercarial shedding but the association of PCR and microscopy techniques point here to natural infection of *G. schirazensis* with *F. hepatica*.

Interestingly, the mean height of the snails positive by microscopy was higher when compared to PCR-positive snails. This could be explained by the fact that younger snails have less developed trematode infections which are more difficult to detect, especially since a sporocyst recently transformed from a miracidium is invisible under the microscope. However, the size of the PCR-positive snails was not statistically different from the size of negative snails.

### Natural prevalences of *Fasciola hepatica*

The prevalence of *F. hepatica* in the intermediate host recorded here (6% with PCR method) is very low when compared to a prevalence of 31.43% (22/70) recorded in *G. cousini* and based on microscopic examination (Ecuador, Machachi) [58]. This latter prevalence is very high for a lymnaeid snail under natural exposure (e.g., [47]). This could be due to the low number of examined snails, high cattle density (10 adult bovines/ha/year), and high fasciolosis prevalence (90%) in the definitive host in the studied farm [58]. In addition, anthropogenic factors involving irrigation of pasture lands by flood or ditches and inappropriate management of grazing cattle are likely to promote infection in both intermediate and definitive hosts in Ecuador [58]. Lower prevalences were observed through microscopy in Brazil where *P. columella* showed infection rates between 0.14% [56] and 5.26% [34] and in Corrientes, Argentina, where a prevalence of 8.8% was recorded [45].

The prevalence through microscopy was statistically higher for the first and second sampling dates. A season effect could have been hypothesized but this is ruled out by the fact that such a difference was not observed with PCR. In Brazil, a study of the *P. columella* population [4] showed that snail density decreased from September to February and increased from March to September with higher metacercaria number on pasture between June–October and March–April. Flooding could be responsible for the observed density reduction during the rainy season [15]. However, seasonal variations are very limited between the wet and dry seasons in the province of Pichincha. The very marked difference of the PCR prevalence between the sites is surprising (site 2 more than eleven times lower). This might be due to a difference in the characteristics of the landscape or in the intermediate host population as was previously recorded [46] and experimentally demonstrated [20, 48, 49]. In such a case, it would be hypothesized that snails at site 1 would be more susceptible to the parasite than snails at site 2. An alternative and non-exclusive hypothesis might be a lower probability of snail infection at site 2.

### *G. schirazensis* role in fasciolosis in Ecuador

Of the different markers used hitherto in lymnaeids, ribosomal DNA ITS-2 and secondarily ITS-1 are the most useful for studies at species level [8]. Within mitochondrial DNA (mtDNA), recent knowledge indicates that these markers should be used with great caution when dealing with lymnaeid species belonging to different genera and even those well separated within the same genus. Of particular concern is the saturation of nucleotide positions, the fact that evolutionary hot spots may be missed, and additionally that there is extensive evidence for mtDNA introgression [53]. The ITS*-2* sequence alignments undoubtedly ascribed the collected lymnaeid snails to *G. schirazensis*. The GenBank sequence [JF272602] came from a voucher deposited in two collections and identified by a specialist [8]. This species was found for the first time in 2009 in Colombia as *Lymnaea* sp. [16] and was also reported in Venezuela, Spain, and La Reunion Island as *Galba* sp. [17], as well as in Iran, Mexico, and Ecuador [8] according to phylogenetic analysis. This species was thus considered as a previously overlooked, highly invasive species [8, 17].

For the first time, our results show the presence of live *F. hepatica* larvae and *F. hepatica* DNA in *G. schirazensis*, which would suggest a potential role of this snail in transmission of fasciolosis. Natural infection studies and experimental infection of *G. schirazensis* with *F. hepatica* were carried out by Bargues and colleagues [8]. None of the 8752 snail specimens collected in the field from 20 localities in eight countries showed cercarial shedding, and the 338 snails experimentally infected did not show the emergence of larval trematodes [8]. Nevertheless, all the experimental infections were allopatric, with few snails (hitherto 10), with only one miracidium, and with a very high mortality rate at 30 days post-infection (hitherto 100%) that did not allow a correct evaluation of the vectorial capacity of *G. schirazensis.* Other experimental infections of *G. schirazensis* with allopatric *F. hepatica* were carried out during five successive snail generations and led to 1.75% (7/400) of snails harboring several immature rediae, rediae containing cercariae, and free cercariae [20]. In our study, *G. schirazensis* was the only freshwater snail species collected in the field, with 8.15% of snails harboring *F. hepatica* DNA and 2.46% of snails with live larvae at site 1, in a farm endemic for fasciolosis. In Colombia and Venezuela, *G. schirazensis* was also the only species found in several very high endemic areas suggesting a role of this species in transmission of fasciolosis [44].

Recently, three alternative developmental pathways of *F. hepatica* were identified in the area where *G. truncatula* (intermediate host of *F. hepatica* in Europe, North Africa, and parts of America) has not been described, particularly in cattle-breeding farms known for high risks of animal fasciolosis [49]. The first pathway involves lymnaeid snails able to sustain complete larval development of the parasite with cercarial shedding if they are infected by miracidia in their first week of life [11, 12]. Nevertheless, experimental infection demonstrated high mortality of the exposed snails, low prevalence, and low cercarial shedding [19]. The second pathway was described during sequential experimental infection of snails with *Calicophoron daubneyi* followed by *F. hepatica*, showing successful infection [1]; this was also observed in some naturally infected snails [2]. A third pathway, probably more sustainable, was recorded during the infection of five successive generations of pre-adult snails originating from parents already infected with this parasite, resulting in a progressive increase in prevalence of snail infection and cercarial emergence [48].

Finally, further studies with sympatric experimental infection, and attempts to obtain cercarial emergence in naturally infected snails and longer sampling campaigns could help elucidate the role of this snail species in fasciolosis in Ecuador. *Galba schirazensis* can currently be considered a potential host of *Fasciola hepatica* in Ecuador.

## Conflict of interest

The authors declare that they have no conflict of interest.

## References

[1] Abrous M, Rondelaud D, Dreyfuss G. 1996 *Paramphistomum daubneyi* and *Fasciola hepatica*: the effect of dual infection on prevalence and cercarial shedding in preadult *Lymnaea glabra*. Journal of Parasitology, 82, 1026–1029. 8973417

[2] Abrous M, Rondelaud D, Dreyfuss G, Cabaret J. 1999 Infection of *Lymnaea truncatula* and *Lymnaea glabra* by *Fasciola hepatica* and *Paramphistomum daubneyi* in farms of central France. Veterinary Research, 30, 113–118. 10081118

[3] Almeyda-Artigas RJ, Bargues MD, Mas-Coma S. 2000 ITS-2 rDNA sequencing of *Gnathostoma* species (Nematoda) and elucidation of the species causing human gnathostomiasis in the Americas. Journal of Parasitology, 86, 537–544. 10864252 10.1645/0022-3395(2000)086[0537:IRSOGS]2.0.CO;2

[4] Amato SB, De Rezende HE, Gomes DC, Da Serra Freire NM. 1986 Epidemiology of *Fasciola hepatica* infection in the Paraiba River Valley, Sao Paulo, Brasil. Veterinary Parasitology, 22, 275–284. 3564331 10.1016/0304-4017(86)90115-9

[5] Andrews JS. 1999 The life cycle of *Fasciola hepatica*, in Fasciolosis. Dalton JP, Editor CABI publishing: Wallingford, Oxon, UK p. 1–29.

[6] Bargues MD, Vigo M, Horak P, Dvorak J, Patzner RA, Pointier JP, Jackiewicz M, Meier-Brook C, Mas-Coma S. 2001 European Lymnaeidae (Mollusca: Gastropoda), intermediate hosts of trematodiases, based on nuclear ribosomal DNA ITS-2 sequences. Infection, Genetics and Evolution, 1, 85–107. 10.1016/s1567-1348(01)00019-3 12798024

[7] Bargues MD, Artigas P, Mera y Sierra RL, Pointier JP, Mas-Coma S. 2007 Characterisation of *Lymnaea cubensis*, *L. viatrix* and *L. neotropica* n. sp., the main vectors of *Fasciola hepatica* in Latin America, by analysis of their ribosomal and mitochondrial DNA. Annals of Tropical Medicine and Parasitology, 101, 621–641. 17877881 10.1179/136485907X229077

[8] Bargues MD, Artigas P, Khoubbane M, Flores R, Gloer P, Rojas-Garcia R, Ashrafi K, Falkner G, Mas-Coma S. 2011 *Lymnaea schirazensis*, an overlooked snail distorting fascioliasis data: genotype, phenotype, ecology, worldwide spread, susceptibility, applicability. PLoS One, 6, e24567. 21980347 10.1371/journal.pone.0024567 PMC3183092

[9] Bargues MD, Gonzalez LC, Artigas P, Mas-Coma S. 2011 A new baseline for fascioliasis in Venezuela: lymnaeid vectors ascertained by DNA sequencing and analysis of their relationships with human and animal infection. Parasites & Vectors, 4, 200. 21999170 10.1186/1756-3305-4-200 PMC3213164

[10] Bernardo C, Carneiro MB, Avelar BR, Donatele DM, Martins IV, Pereira MJ. 2011 Prevalence of liver condemnation due to bovine fasciolosis in Southern Espirito Santo: temporal distribution and economic losses. Brazilian Journal of Veterinary Parasitology, 20, 49–53. 21439232 10.1590/s1984-29612011000100010

[11] Boray JC. 1969 Experimental fascioliasis in Australia. Advances in Parasitology, 7, 95–210. 4935272 10.1016/s0065-308x(08)60435-2

[12] Boray JC. 1978 The potential impact of exotic *Lymnaea* spp*.* on fascioliasis in Australasia. Veterinary Parasitology, 4, 127–141.

[13] Caron Y, Rondelaud D, Losson B. 2008 The detection and quantification of a digenean infection in the snail host with special emphasis on *Fasciola* sp. Parasitology Research, 103, 735–744. 18574594 10.1007/s00436-008-1086-1

[14] Caron Y, Righi S, Lempereur L, Saegerman C, Losson B. 2011 An optimized DNA extraction and multiplex PCR for the detection of *Fasciola* sp. in lymnaeid snails. Veterinary Parasitology, 178, 93–99. 21242033 10.1016/j.vetpar.2010.12.020

[15] Coelho LH, Lima WS. 2003 Population dynamics of *Lymnaea columella* and its natural infection by *Fasciola hepatica* in the State of Minas Gerais, Brazil. Journal of Helminthology, 77, 7–10. 12590657 10.1079/JOH2002138

[16] Correa AC, Escobar JS, Durand P, Renaud F, David P, Jarne P, Pointier JP, Hurtrez-Bousses S. 2010 Bridging gaps in the molecular phylogeny of the Lymnaeidae (Gastropoda: Pulmonata), vectors of fascioliasis. BMC Evolutionary Biology, 10, 381. 21143890 10.1186/1471-2148-10-381 PMC3013105

[17] Correa AC, Escobar JS, Noya O, Velasquez LE, Gonzalez-Ramirez C, Hurtrez-Bousses S, Pointier JP. 2011 Morphological and molecular characterization of Neotropic Lymnaeidae (Gastropoda: Lymnaeoidea), vectors of fasciolosis. Infection, Genetics and Evolution, 11, 1978–1988. 10.1016/j.meegid.2011.09.003 21968212

[18] Cucher MA, Carnevale S, Prepelitchi L, Labbe JH, Wisnivesky-Colli C. 2006 PCR diagnosis of *Fasciola hepatica* in field-collected *Lymnaea columella* and *Lymnaea viatrix* snails. Veterinary Parasitology, 137, 74–82. 16427203 10.1016/j.vetpar.2005.12.013

[19] Dreyfuss G, Abrous M, Rondelaud D. 2000 The susceptibility of *Lymnaea fuscus* to experimental infection with *Fasciola hepatica*. Journal of Parasitology, 86, 158–160. 10701582 10.1645/0022-3395(2000)086[0158:TSOLFT]2.0.CO;2

[20] Dreyfuss G, Correa AC, Djuikwo-Teukeng FF, Novobilsky A, Höglund J, Pankrác J, Kasny M, Vignoles P, Hurtrez-Boussès S, Pointier JP, Rondelaud D. 2014 Differences of compatibility for a Colombian population of *Galba* sp. between *Fascioloides magna* and *Fasciola hepatica*. Journal of Helminthology, 7, 1–7. 10.1017/S0022149X14000509 25000491

[21] Egas-Dávila R, Villota-Burbano M, Celi-Erazo M, Ron-Román J, Proaño-Perez F, Rodríguez-Hidalgo R, Benítez-Ortis W. 2006 Determinación de la prevalencia de Fasciola hepatica en bovinos sacrificados en el camal municipal de Machachi: Trazabilidad de los animales positivos, Thesis, Universidad Central del Ecuador: Quito, Ecuador.

[22] Espinoza JR, Terashima A, Herrera-Velit P, Marcos LA. 2010 Human and animal fascioliasis in Peru: impact in the economy of endemic zones. Peruvian Review of Experimental Medicine and Public Health, 27, 604–612. 10.1590/s1726-46342010000400018 21308203

[23] Esteban JG, Flores A, Angles R, Mas-Coma S. 1999 High endemicity of human fascioliasis between Lake Titicaca and La Paz valley, Bolivia. Transactions of the Royal Society of Tropical Medicine and Hygiene, 93, 151–156. 10450437 10.1016/s0035-9203(99)90289-4

[24] Gozalbo M, Trueba G, Fornasini M, Fuentes MV, Bargues MD, Esteban JG, Mas-Coma MS. 2004 Coproparasitological survey in schoolchildren from the community of Planchaloma (Province de Cotopaxi, Ecuador), in Multidisciplinarity for Parasites, Vectors and Parasitic Diseases, IX European Multicolloquium of Parasitology (EMOP 9), Programme and Abstracts. Mas-Coma S, Bargues MD, Esteban JG, Valero MA, Editors J. Aguilar S.L.: Valencia, Spain, Abstract No. 874: 447 (Spanish version in Enfermedades Emergentes, 6, 3, No. 84: 170).

[25] Gutierrez A, Vazquez AA, Hevia Y, Sanchez J, Correa AC, Hurtrez-Boussès S, Pointier JP, Theron A. 2011 First report of larval stages of *Fasciola hepatica* in a wild population of *Pseudosuccinea columella* from Cuba and the Caribbean. Journal of Helminthology, 85, 109–111. 20637143 10.1017/S0022149X10000350

[26] Hall TA. 1999 BioEdit: a user-friendly biological sequence alignment editor and analysis program for Windows 95/98/NT. Nucleic Acids Symposium Series, 41, 95–98.

[27] Hillyer GV, Soler de Galanes M, Rodriguez-Perez J, Bjorland J, Silva de Lagrava M, Ramirez Guzman S, Bryan RT. 1992 Use of the Falcon assay screening test – enzyme-linked immunosorbent assay (FAST-ELISA) and the enzyme-linked immunoelectrotransfer blot (EITB) to determine the prevalence of human fascioliasis in the Bolivian Altiplano. American Journal of Tropical Medicine and Hygiene, 46, 603–609. 1599055 10.4269/ajtmh.1992.46.603

[28] Hurtrez-Boussès S, Meunier C, Durand P, Renaud F. 2001 Dynamics of host-parasite interactions: the example of population biology of the liver fluke (*Fasciola hepatica*). Microbes and Infection, 3, 841–849. 11580979 10.1016/s1286-4579(01)01442-3

[29] Kaplan RM, Dame JB, Reddy GR, Courtney CH. 1995 A repetitive DNA probe for the sensitive detection of *Fasciola hepatica* infected snails. International Journal for Parasitology, 25, 601–610. 7635638 10.1016/0020-7519(94)00159-l

[30] Keiser J, Utzinger J. 2005 Emerging foodborne trematodiasis. Emerging Infectious Diseases, 11, 1507–1514. 16318688 10.3201/eid1110.050614 PMC3366753

[31] Krämer F, Schnieder T. 1998 Sequence heterogeneity in a repetitive DNA element of *Fasciola*. International Journal for Parasitology, 28, 1923–1929. 9925273 10.1016/s0020-7519(98)00162-3

[32] Lounnas M, Vázquez AA, Alda P, Sartori K, Pointier JP, David P, Hurtrez-Boussès S. 2017 Isolation, characterization and population-genetic analysis of microsatellite loci in the freshwater snail *Galba cubensis* (Lymnaeidae). Journal of Molluscan Studies, 83, 63–68.

[33] Mas-Coma S. 2007 *Lymnaea cousini* (Gastropoda: Lymnaeidae) as transmitter of fascioliasis. Memórias do Instituto Oswaldo Cruz, 102, 241–243. 17426894 10.1590/s0074-02762007005000023

[34] Oliveira SM, Fujii TU, Sposito Filha E, Martins A. 2002 Ocorrência de *Lymnaea columella* Say, 1817 infectada naturalmente por *Fasciola hepatica* (Linnaeus, 1758), no Vale do Ribeira, São Paulo, Brasil. Arquivos do Instituto de Biologia, 15, 37–69.

[35] Paraense WL. 1976 *Lymnaea viatrix*: a study of topotypic specimens (Mollusca: Lymnaeidae). Revista Brasileira de Biologia, 36, 419–428.

[36] Paraense W. 1982a *Lymnaea rupestris* sp. n. from southern brazil (Pulmonata: lymnaeidae). Memórias do Instituto Oswaldo Cruz, 77, 437–443.

[37] Paraense WL. 1982b *Lymnaea viatrix* and *Lymnaea columella* in the neotropical region: a distribution outline. Memórias do Instituto Oswaldo Cruz, 77, 181–188.

[38] Paraense WL. 1984 *Lymnaea diaphana*: a study of topotypic specimens (Pulmonata: Lymnaeidae). Memórias do Instituto Oswaldo Cruz, 79, 75–81.

[39] Paraense WL. 1986 *Lymnaea columella*: two new Brazilian localities in the state of Amazonas and Bahia. Memórias do Instituto Oswaldo Cruz, 81, 121–123.

[40] Paraense WL. 1995 *Lymnaea cousini* Jousseaume, 1887, from Ecuador (Gastropoda, Lymnaeidae). Memórias do Instituto Oswaldo Cruz, 90, 605–609.

[41] Paraense WL. 2004 Planorbidae, Lymnaeidae and Physidae of Ecuador (*Mollusca*: *Basommatophora*). Memórias do Instituto Oswaldo Cruz, 99, 357–362. 15322623 10.1590/s0074-02762004000400003

[42] Petrie A, Watson P. 2006 Statistics for Veterinary and Animal Science, 2nd edn Wiley: Ames.

[43] Pointier JP, Cazzaniga NJ, Gonzalez-Salas C, Gutierrez A, Arenas JA, Bargues MD, Mas-Coma S. 2006 Anatomical studies of sibling species within Neotropical lymnaeids, snail intermediate hosts of fascioliasis. Memórias do Instituto Oswaldo Cruz, 101, 431–435. 16951816 10.1590/s0074-02762006000400015

[44] Pointier JP, González C, Noya O, Alarcón de Noya B. 2015 Family Lymnaeidae, in Freshwater Molluscs of Venezuela and their medical and veterinary importance. Pointier JP, Editor Harxheim, Germany p. 101–124.

[45] Prepelitchi L, Kleiman F, Pietrokovsky SM, Moriena RA, Racioppi O, Alvarez J, Wisnivesky-Colli C. 2003 First report of *Lymnaea columella* Say, 1817 (Pulmonata: Lymnaeidae) naturally infected with *Fasciola hepatica* (Linnaeus,1758) (Trematoda: Digenea) in Argentina. Memórias do Instituto Oswaldo Cruz, 98, 889–891. 14762513 10.1590/s0074-02762003000700005

[46] Rondelaud D. 1993 Variabilité interpopulationnelle de l’infestation fasciolienne chez le mollusque *Lymnaea truncatula* Müller. Influence du contact préalable de la population avec le parasite. Bulletin de la Société Française de Zoologie, 118, 185–193.

[47] Rondelaud D, Vignoles P, Dreyfuss G. 2009 La limnée tronquée, un mollusque d’intérêt médical et vétérinaire. PULIM: Limoges.

[48] Rondelaud D, Djuikwo-Teukeng FF, Vignoles P, Dreyfuss G. 2014 *Lymnaea glabra*: progressive increase in susceptibility to *Fasciola hepatica* through successive generations of experimentally-infected snails. Journal of Helminthology, 16, 1–6. 10.1017/S0022149X14000169 24735873

[49] Rondelaud D, Titi A, Vignoles P, Mekroud A, Dreyfuss G. 2014 Adaptation of *Lymnaea fuscus* and *Radix balthica* to *Fasciola hepatica* through the experimental infection of several successive snail generations. Parasites & Vectors, 7, 296–302. 24986589 10.1186/1756-3305-7-296 PMC4090179

[50] Samadi S, Roumegoux A, Bargues MD, Mas-Coma MS, Yong M, Pointier JP. 2000 Morphological studies of Lymnaeid snails from the human fascioliasis endemic zone of Bolivia. Journal Molluscan Studies, 66, 31–44.

[51] Schell SC. 1985 Handbook of trematodes of North America north of Mexico. Moscow, Idaho, USA: University Press of Idaho, ISBN: 0-89301-095-2.

[52] Schweizer G, Braun U, Deplazes P, Torgerson PR. 2005 Estimating the financial losses due to bovine fasciolosis in Switzerland. Veterinary Record, 157, 188–193. 16100368 10.1136/vr.157.7.188

[53] Toews DP, Brelsford A. 2012 The biogeographiy of mitochondrial and nuclear discordance in animals. Molecular Ecology, 21, 3907–3930. 22738314 10.1111/j.1365-294X.2012.05664.x

[54] Torgerson P, Claxton J. 1999 Epidemiology and control, in Fasciolosis. Dalton JP, Editor. CABI Publishing: Wallingford, Oxon, UK.

[55] Trueba G, Guerrero T, Fornasini M, Casariego I, Zapata S, Ontaneda S, Vasco L. 2000 Detection of *Fasciola hepatica* infection in a community located in the Ecuadorian Andes. American Journal of Tropical Medicine and Hygiene, 62, 518. 11220770 10.4269/ajtmh.2000.62.518

[56] Ueta MT. 1980 Ocorrência de infecção natural de *Fasciola hepatica* Linnaeus, 1758 em *Lymnaea columella* Say, 1817 no Vale do Paraiba, SP. Brazilian Review of Public Health, 14, 230–233. 10.1590/s0034-89101980000200010 7221470

[57] Utaaker KS, Robertson LJ. 2015 Climate change and foodborn transmission of parasites: A consideration of possible interactions and impacts for selected parasites. Food Research International, 68, 16–23.

[58] Villavicencio A, Vasconcellos MC. 2005 First report of *Lymnaea cousini* Jousseaume, 1887 naturally infected with *Fasciola hepatica* (Linnaeus, 1758) (Trematoda: Digenea) in Machachi, Ecuador. Memórias do Instituto Oswaldo Cruz, 100, 735–737. 16410961 10.1590/s0074-02762005000700010

